# Optimal INR level for warfarin therapy after mechanical mitral valve replacement

**DOI:** 10.1186/s12872-019-1078-3

**Published:** 2019-04-25

**Authors:** Itthidet Kamthornthanakarn, Rungroj Krittayaphong

**Affiliations:** Division of Cardiology, Department of Medicine, Faculty of Medicine Siriraj Hospital, Mahidol University, 2 Wanglang Road, Bangkoknoi, Bangkok, 10700 Thailand

**Keywords:** Thailand, Optimal INR, Warfarin, Mechanical mitral valve replacement, Thromboembolism, Bleeding

## Abstract

**Background:**

Data are scarce regarding the optimal international normalized ratio (INR) in Thai patients who require warfarin therapy after mechanical mitral valve replacement. Accordingly, the aim of this study was to identify the optimal INR level for warfarin therapy after mechanical mitral valve replacement in Thai patients.

**Methods:**

This is a retrospective cohort study design. We retrospectively reviewed the medical records of mechanical mitral valve replacement patients who received warfarin therapy at Siriraj Hospital. INR range was classified into 6 groups (< 2, 2.0–2.4, 2.5–2.9, 3.0–3.4, 3.5–4.5, and > 4.5). The optimal INR level was defined as the level with the lowest incidence density of thromboembolic or hemorrhagic complications.

**Results:**

Two hundred patients were included and followed over a period of 707.81 patient-years. Mean duration of follow-up was 3.53 ± 1.27 years. Eleven patients experienced 13 thromboembolic events (3.42 per 100 patient-years), and 12 patients experienced 18 total bleeding events (5.50 per 100 patient-years). Intracranial bleeding occurred in 3 patients (2.62 per 100 patient-years). The percentage of patient time spent within INR 2.5–3.4, INR < 2.5, and INR > 3.4 was 41.96, 54.04, and 4%, respectively. The overall event rate was lowest in the 2.0 to 3.4 INR range. Statistically significant differences were observed between INR 2.3 to 4 and < 2 (*p* < 0.001) and between INR 2.3 to 4 and > 3.4 (*p* < 0.001).

**Conclusions:**

The optimal INR level was within the range of 2.0 to 3.4 in our cohort of Thai mechanical mitral valve replacement patients.

## Background

Lifelong oral anticoagulation therapy for prevention of thromboembolic events is recommended in all patients who undergo mechanical heart valve replacement [1–3]. However, bleeding complications that result from excess dose of anticoagulants adversely impacts patients’ quality of life and can cause unnecessary morbidity and mortality [4]. Risk of thromboembolism and bleeding is commensurate with the level of anticoagulation. Current American and European clinical guidelines recommend a higher international normalized ratio (INR) for anticoagulant therapy after mechanical mitral valve replacement, because higher rates of thromboembolic complications were reported when the mechanical valve was in the mitral position, as compared to when the mechanical valve was in the aortic position [5–8]. A target INR range of 2.5–3.5 is the current recommendation in patients who have undergone mechanical mitral valve replacement [5–7]. These data were based on results from studies conducted in Western countries; however and according to our review of the literature, data regarding the safety of warfarin in Asian populations remain insufficient. Previous data have shown that Asian population with atrial fibrillation who received warfarin has an increased risk of intracranial hemorrhage up to 4 times compared to Caucasians [9]. Asian population had a greater proportion of intracerebral bleeding as a stroke subtype when compared to Caucasians [10]. There is no clear explanation for the increased risk of intracerebral bleeding in Asian population [10]. The few studies that were conducted in Asian populations who received mechanical heart valve replacement generally recommended a lower INR level as being appropriate for anticoagulant therapy [11–13]. However, the currently available data from Thai population are insufficient for defining the optimal INR level for anticoagulant therapy in Thai mechanical valve recipients. Accordingly, the aim of this study was to identify the optimal INR level for warfarin therapy after mechanical mitral valve replacement in Thai patients.

## Methods

### Study design

This is a retrospective cohort study design. The data were retrospectively extracted. Follow-up data were also extracted for the average of 3.53 ± 1.27 years.

### Study area and site

This study was conducted at Siriraj Hospital, Mahidol University, Bangkok, Thailand. Siriraj Hospital is the Thailand’s largest university-based national tertiary referral center.

### Study population and sample size calculation

We studied patients who underwent mechanical mitral valve replacement and received warfarin therapy at Siriraj Hospital during the 2011 to 2015.

We calculated sample size based on the results of a previous publication by Marieke Torn, et al., 2009 [14]. They reported the incidence of thromboembolic and bleeding events in the < 2, 2.0–2.4, 2.5–2.9, 3.0–3.4, 3.5–4.5, and > 4.5 INR groups to be 0.319, 0.067, 0.02, 0.025, 0.033, and 0.247, respectively. We calculated sample size by G group Chi-square test comparing proportions in C Categories formula by nQuery program (Statistical Solutions Ltd., Cork, Ireland). From the estimation of proportion of patients in each INR group, we calculates the ratio of ni/n_1_ assuming that n_1_ which is the number of patients in the group with the lowest INR (INR < 2) = 1. The calculated ratio of n for the 6 INR groups were 1: 6: 4.2: 4: 4: and 0.8. The average proportion of thromboembolism and bleeding outcome from the calculation was 0.062 and variance of proportion was 0.005. By using the proportion of outcome at 0.062, variance of 0.005, with the power of 90% (a type II error of 0.10), a type I error of 0.05, The sample size of all groups was 192 patients. We then proposed the total sample size of 200 patients.

### Ethics approval

The protocol for this study was approved by the Siriraj Institutional Review Board (SIRB), Faculty of Medicine Siriraj Hospital, Mahidol University, Bangkok, Thailand. The Institutional Review Board of Faculty of Medicine Siriraj Hospital approved the study after determining that the study met the definition of research not requiring informed consent.

### Study procedure

After the IRB approval, we requested the data of potential study patients from the hospital data department and from the cardiothoracic department. We reviewed the data from the electronic medical record to screen for inclusion and exclusion criteria and recorded data in the case record form. We recorded the data from case record form into SPSS Statistics version 18.0 (SPSS, Inc., Chicago, IL, USA) for statistical analysis. The statistician cleaned data and query was sent to the investigators for verification.

### Sampling technique

All patients who underwent mechanical mitral valve replacement and received warfarin therapy at Siriraj Hospital during the 2011 to 2015 were included in this study.

### Inclusion and exclusion criteria

Inclusion criteria were Thai patients aged ≥18 years who underwent mechanical mitral valve replacement and received warfarin. Patients with one or more of the following were excluded: 1) history of thromboembolic or bleeding complications; 2) contraindications to oral anticoagulation therapy; 3) platelet count < 100,000/mm3 during bleeding events; 4) thromboembolic events from heparin-induced thrombocytopenia, myeloproliferative disorders, or hyperviscosity syndrome; or, 5) did not have follow-up data.

### Data collection tool and data collection

We collected demographic data, clinical data, types of mechanical valve, INR levels, and warfarin dose. INR level was classified into the following 6 groups for analysis: < 2, 2.0–2.4, 2.5–2.9, 3.0–3.4, 3.5–4.5, and > 4.5. The INR-specific incidence density was calculated as the ratio of the number of thromboembolic or bleeding events that occurred in each INR group to the total amount of time (patient-years) that each patient stayed in each INR group, according to the method described by Frits R Rosendaal, et al., 1993 [15]. Assignment of an event to an INR group was made using the first INR during the event or the most recent INR level within 7 days prior to the occurrence of the thromboembolic or bleeding event.

The time that each patient stayed in each INR group was calculated by taking the duration between when a patient was in one INR group to when that same patient had an INR score that was in another INR group. That total duration was then divided in half, and half of the time was allocated to the prior INR group and the other half of the time was allocated to the new INR group. For example, if a patient had an INR of 2.2 that became 2.7 twelve weeks later, the time in the 2.0–2.4 INR group was 6 weeks and the time in the 2.5–2.9 INR group was 6 weeks.

Thromboembolic events consisted of ischemic stroke, peripheral thromboembolism, and prosthetic valve thrombosis. Ischemic stroke was defined as sudden onset neurologic deficit lasting less than or greater than 24 h, and confirmed by computed tomography (CT) scan with or without significant carotid artery stenosis from carotid Doppler ultrasonography or carotid bruit. Peripheral thromboembolism was diagnosed as sudden onset peripheral ischemia, proven by duplex scanning, angiography, surgery, or autopsy.

Bleeding complications included both clinically significant and minor bleeding events. Clinically significant bleeding was defined as 1) intracranial, spinal, subdural hematoma, and/or subarachnoid hemorrhage; 2) gastrointestinal bleeding that required at least 2 units of blood transfusion or that required hospital admission for observation and/or treatment; 3) hemoptysis that required hospital admission for observation and/or treatment; or, 4) gross hematuria that required continuous bladder irrigation or that resulted in the patient becoming hemodynamically compromised. Minor bleeding was defined as bleeding events other than clinically significant bleeding. We modified the criteria of clinically significant bleeding following some criteria of the International Society of Thrombosis and Haemostasis (ISTH) [16], and some from the Bleeding Academic Research Consortium (BARC) [17] to include bleeding event requiring medical attention which has been used in major clinical study [18].

Regarding the dead patients, when the death were related to ischemic stroke we count as ischemic stroke, when it was related to severe bleeding, we counted as clinically significant bleeding. But when the death is not related to ischemic stroke or bleeding, we did not count as an outcome.

### Statistical analysis

SPSS Statistics version 18.0 (SPSS, Inc., Chicago, IL, USA) was used for all statistical analyses. Categorical data, such as gender and comorbid diseases, are presented as frequency and percentage. Continuous variables, such as age, left ventricular ejection fraction (LVEF), left atrium (LA) volume index, and LA diameter, are expressed as mean ± standard deviation. Kolmogorov Smirnov test was used to test the normality of the data distribution. All continuous variables inn this study such as age, LVEF, LA volume, LA diameter, and INR were normally distributed. The optimal INR level was determined by comparing the incidence density among all 6 INR groups. The incidence density of thromboembolic and bleeding complications was compared using chi-square test. The optimal INR level was defined as the lowest incidence density of thromboembolic or hemorrhagic complications. Concerning the comparing repeated measure INR data, since the number of INR tests during follow-up of the study population varied, it may not be suitable to use repeated measure ANOVA to run the analysis. For repeated measure ANOVA analysis, the number of repeated measure data should be the same to prevent excluding patients’ data from analysis. Linear mixed model (fixed effect) is the preferred test to compare the repeated measurement INR levels related outcome measures at time. A *p*-value less than 0.05 was regarded as being statistically significant for all tests.

## Results

Two hundred patients were included and followed till 707 patient-years. Mean duration of follow-up was 3.53 ± 1.27 years. Mean age of patients was 50.30 ± 10.9 years, with a gender proportion breakdown of 116 (58%) females and 84 (42%) males. Two-thirds (70.5%) of patients had rheumatic valve disease. All patients received bileaflet mechanical valves. Left atrial appendage closure was performed during surgery in 19 patients (9.5%). Twenty-seven (13.5%) patients had concomitant aortic valve replacement (AVR), and 147 (73.50%) of patients had atrial fibrillation. Among patients with atrial fibrillation, only 4 (2.7%) were paroxysmal atrial fibrillation in nature. The number was too small to make any comparison for the incidence of thromboembolic events. Hypertension was the most common comorbidity (26.5%). Seven (3.5%) patients received concomitant aspirin. Baseline demographic characteristics, clinical characteristics, and laboratory parameters of Thai mechanical mitral valve replacement patients are shown in Table 1.

**Table 1:** Baseline characteristics of Thai mechanical mitral valve replacement patients

Characteristics	(*N* = 200)
Age (years), mean ± SD (range)	50.30 ± 10.90 (18–75)
Female gender, n (%)	116 (58%)
Atrial fibrillation, n (%)	147 (73.5%)
Comorbidity, n (%)	
-Diabetes mellitus	17 (8.5%)
-Hypertension	53 (26.5%)
-Chronic kidney disease	13 (6.5%)
-Dyslipidemia	41 (20.5%)
-Coronary artery disease	21 (10.5%)
Echocardiographic findings	
-LVEF (%), mean ± SD	63.20 ± 10.60
-LVEF < 40%, n (%)	7 (3.5%)
-LA volume index (ml/m2), mean ± SD	115.79 ± 75.72
-LA diameter (mm), mean ± SD	58.88 ± 10.82
-LA thrombus, n (%)	5 (2.5%)
Concomitant aspirin, n (%)	7 (3.5%)
Pathology of valve disease, n (%)	
-Rheumatic	141 (70.5%)
-Ruptured chordae tendinae	35 (17.5%)
-Prolapsed	22 (11%)
-Others	2 (1%)
Mechanical valve, n (%)	
-Bileaflet valve	200 (100%)
Concomitant AVR, n (%)	27 (13.5%)

Abbreviations: *SD* standard deviation, *LVEF* left ventricular ejection fraction, *LA* left atrium, *AVR* aortic valve replacement

**Fig. 1:**
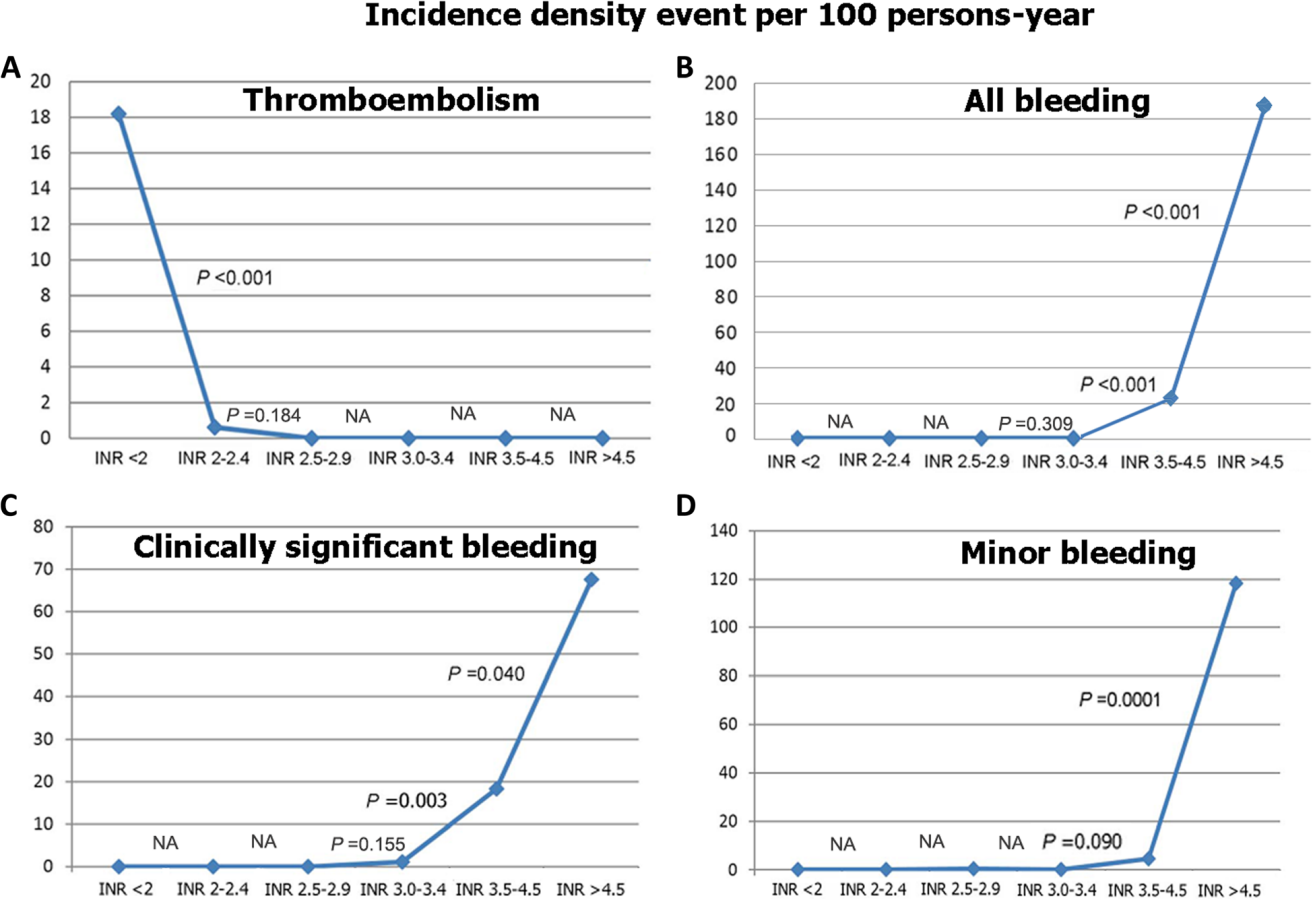
Incidence density of thromboembolic events (**a**), all bleeding (**b**), clinically significant (**c**) and minor bleeding (**d**) per 100 person-years at each INR level. Abbreviation: INR, international normalized ratio

A total of 31 thromboembolism and bleeding events (4.38 per 100 patient-years) were documented during the follow-up period (Table 2). Eleven patients experienced 13 thromboembolic events (3.42 per 100 patient-years), and 12 patients experienced 18 total bleeding events (5.50 per 100 patient-years). Intracranial bleeding occurred in 3 patients (2.62 per 100 patient-years). There was one patient who died from intracerebral hemorrhage and another patient who died from a large cerebral infarction. The percentage of patient time spent within INR 2.5–3.4, INR < 2.5, and INR > 3.4 was 41.96, 54.04, and 4%, respectively. We analyzed and compared the incidence density of thromboembolic (Fig. 1a), total bleeding, clinically significant bleeding, and minor bleeding events (Fig. 1b, c, d) between INR groups using chi-square test. The average dose of warfarin in the study sample was 25.6 ± 9.5 mg per week. The frequency of controlled INR of 2.5–3.5 based on standard recommendation was 40.4 ± 16.3%. Average frequency of INR monitoring was once every 3.1 ± 1.0 month. One-hundred ninety-one patients (91.5%) had warfarin dose optimization. The frequency of based on the INR test was 3.31.8 times during the study period. Analysis by linear mixed models (fixed effect) to study the difference in INR levels in patients with and without events at time indicates that the average INR levels of bleeding, minor bleeding and major bleeding group was more than non-event group (*p* < 0.001) but the average INR levels of thromboembolism group was lower than non-event group (*p* < 0.001)(Table 3).

**Table 2:** Thromboembolic and bleeding events documented during the follow-up period

Events	Total (*N* = 200)	Per 100 person-years
Thromboembolic events, n (%)	13 (6.5%)	3.42
-Stroke	9 (4.5%)	
-TIA	2 (1.0%)	
-Valve thrombosis	2 (1.0%)	
Bleeding events, n (%)	18 (9.0%)	5.50
-Clinically significant	9 (4.5%)	
-Minor	9 (4.5%)	
Total events, n (%)	31 (15.5%)	4.38

Abbreviation: *TIA* transient ischemic attack

**Table 3:** The effect of repeated INR level and event outcome measures at time

	Mean INR	Standard error of mean	Mean difference (95% CI)	Effect Estimate (Beta)	Effect Standard Error	*P*-value
Thromboembolic	1.69	0.11	−0.86	−0.88	0.19	< 0.001
No Thromboembolic	2.55	0.01	(−1.24 to − 0.49)			
Bleeding	5.05	0.53	2.53	2.52	0.154	< 0.001
No bleeding	2.53	0.01	(2.22 to 2.83)			
Major	4.95	0.76	2.41	2.41	0.22	< 0.001
No major	2.54	0.01	(1.97 to 2.85)			
Minor	5.16	0.79	2.63	2.61	0.22	0.002
No minor	2.54	0.01	(2.19 to 3.06)			
Follow-up time point				0.004	0.003	0.121

The interaction effect between Thromboembolic, bleeding, minor bleeding and major bleeding event and time was non-significant (P-value > 0.05)

**Fig. 2:**
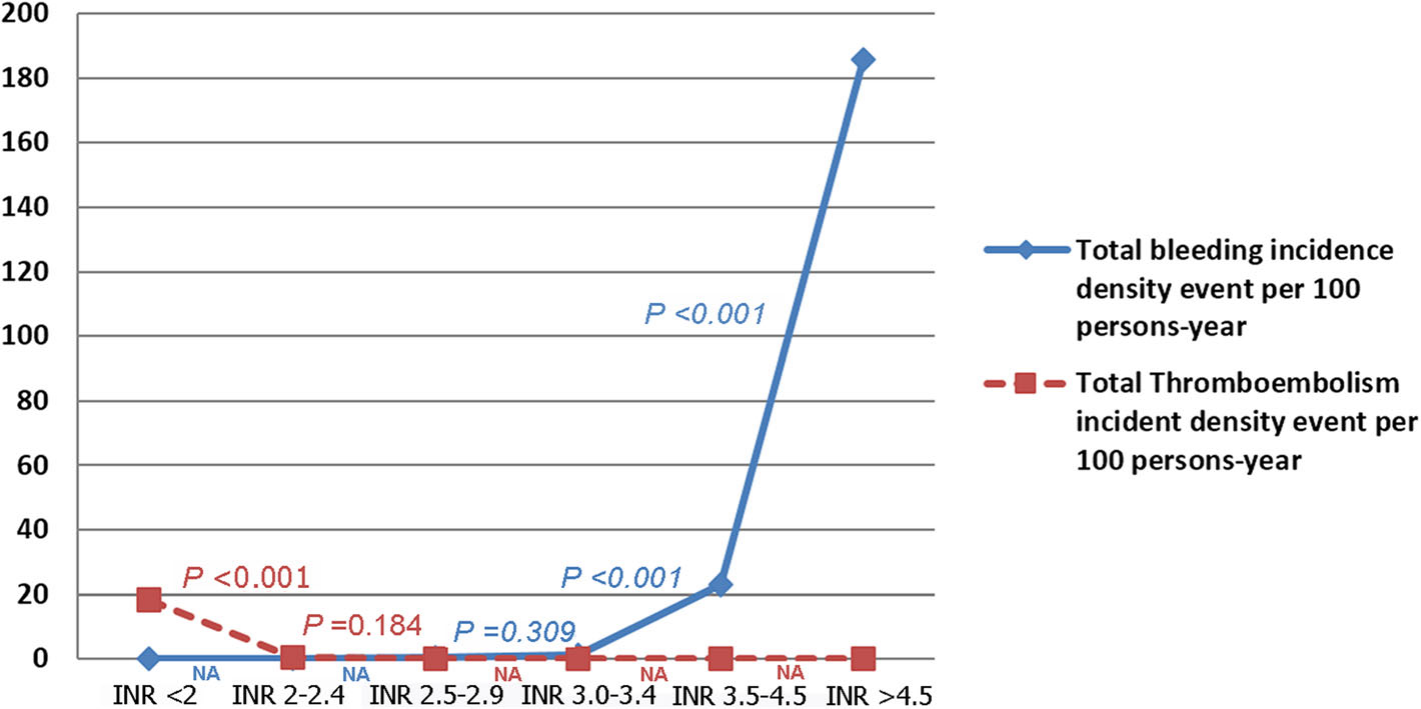
Incidence density of thromboembolic events and total bleeding events per 100 person-years at each INR level. Abbreviation: INR, international normalized ratio

**Fig. 3:**
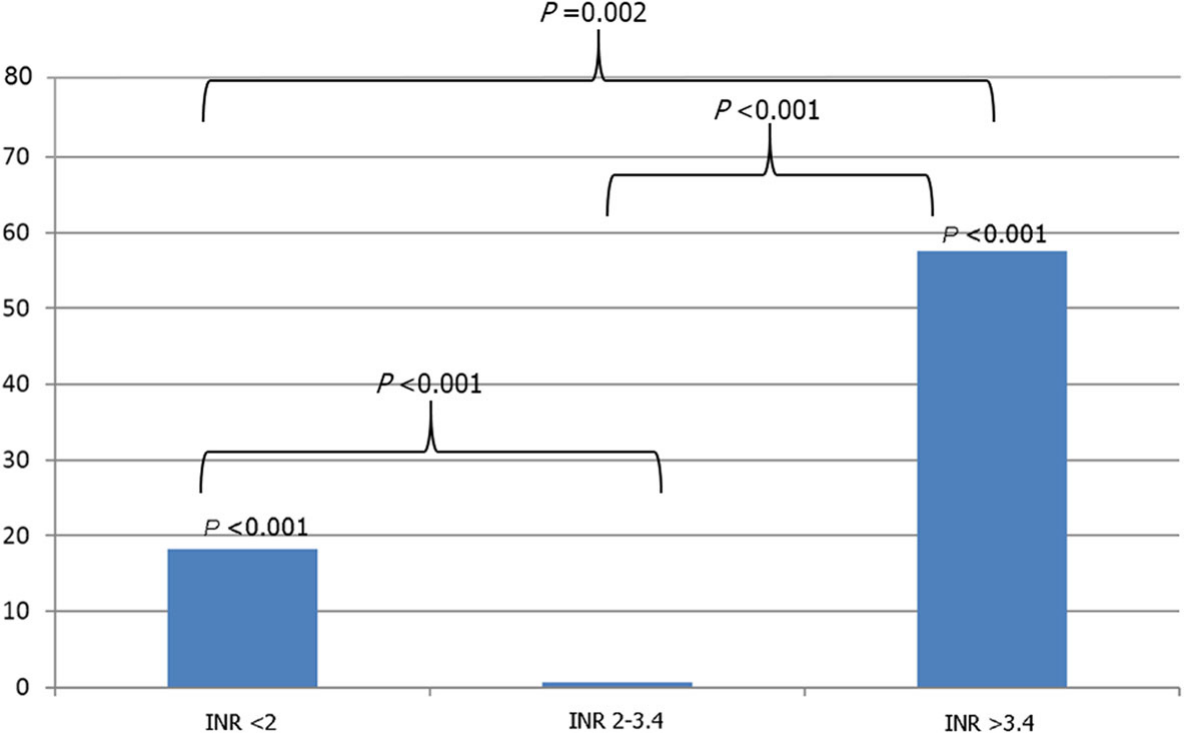
Incidence density of thromboembolic events and total bleeding events per 100 person-years at INR level 2.0–3.4, as compared with the incidence density at lower and higher INR levels. Abbreviation: INR, international normalized ratio

The optimal INR range in Thai patients with mechanical mitral valve replacement to be 2.0–3.4. An INR level greater than 3.4 significantly increased the incidence density of total bleeding events (*p* = 0.001; rate ratio 21.18, 95% confidence interval 6.88–49.46), whereas an INR level less than 2 significantly increased the incidence density of thromboembolic events (*p* < 0.001; rate ratio 29.00, 95% confidence interval 6.33–269.26). Accordingly, we concluded that the optimal INR level was 2.0–3.4, as show in Fig. 2. The overall event rate was lowest in the 2.0 to 3.4 INR range, with statistically significant differences being observed between INR 2.3 to 4 and < 2 (*p* < 0.001) and between INR 2.3 to 4 and > 3.4 (*p* < 0.001) (Fig. 3).

## Discussion

This is the first study in INR levels for warfarin therapy in Thai patients with mechanical mitral valve replacement. This study found that Thai patients with mechanical mitral valve replacement required lower INR levels for warfarin therapy than Western patients according to the clinical guidelines [5–7]. However, the results of this study are consistent with the results of studies in mechanical mitral valve replacement patients from China and Japan [11–13]. These observed disparities between Asian and Western populations may be attributable race-related differences.

In a report from China, Xin-Min Zhou, et al., 2005 [11] described a rate of 5.83 per 100 patient-years for bleeding events, and 0.26 per 100 patient-years for thromboembolic events in patients who received a Carbomedics valve replacement. Compared to our data, Zhou’s study included both mechanical mitral and aortic valve, had a shorter time of follow-up (377 vs 706 patient-years), and had fewer thromboembolic event (1 event vs 13 events). They concluded that low-intensity oral anticoagulant (1.4–2.0) in patients with mechanical valves could effectively prevent thromboembolism and markedly decrease bleeding events. The optimal INR range in our study was 2.0–3.4 which is higher than Zhou’s and may be related to the study population who was post mitral valve replacement in all cases in our study but mitral or aortic valve replacement for their study.

Heinrich Koertke, et al., 2010 [19] followed 1137 German patients in a prospective randomized multicenter trial and demonstrated the efficacy and safety of very low, self-managed INR doses in patients with aortic valve replacement (INR target value: 2.0, range: 1.6–2.1), and in patients with mitral valve or double valve replacement (INR target value: 2.3, range: 2.0–2.5). The results of this study is in a similar direction to our study and indicate that even in Caucasians, a carefully managed strategy can reduce the INR target in patients after mechanical valve replacement. In France, the AREVA study reported an incidence of thromboembolic complications that was similar between patients with a target INR range of 2.0–3.0 and patients with a target INR range of 3.0–4.5; however, there were fewer bleeding complications in the low-dose group [20]. The study population of our study were different from the aforementioned studies [11, 19, 20]. They enrolled both mitral and aortic valve replacement but we enrolled only patients with mechanical mitral valve. Majority of patients enrolled in AREVA study were those with aortic valve replacement. Patients with aortic valve replacement usually require a lower INR target than those with mitral valve replacement [21, 22].

Previous Asian studies reported a higher incidence of bleeding complications among Asian warfarin users than among Western users [9]. The overall event rate for ischemic stroke or TIA in the present study was 5.5%, while the overall event rate for bleeding complications was 9% (4.5% clinically significant and 4.5% minor). Therefore, the overall rate of bleeding events found in this study was similar to the rates reported from previously published Asian and Western studies but the rate of thromboembolic event in our study were higher [11–13, 19, 20]. The higher rate of ischemic stroke may be related to a suboptimal TTR control in our population compared to other studies [23, 24]. Doctors may be fear of bleeding and keep a relatively low INR levels. It has been reported from the GARFIELD AF registry that TTR of Asian population is lower than Caucasians [25]. But despite the lower TTR, Asian population had a higher rate of major bleeding and intracerebral hemorrhage compared to Caucasians [10]. The higher ischemic stroke rate in our study as compared to study from China [11] may be due to the study population. We enrolled patients with mechanical mitral valve prosthesis whereas study from China enrolled both mitral and aortic valve prosthesis. Patients with aortic valve prosthesis had a lower thrombotic complication and required a lower INR target compared to those with mitral valve prosthesis [21, 22].

From the results of the present study, we concluded that 2.0–3.4 is the INR range at which the lowest incidence of thromboembolism and bleeding complications should be anticipated. When we combined the 6 INR groups into 3 INR groups (INR less than 2.0, INR 2.0–3.4, and INR greater than 3.4), INR 2.0–3.4 had significantly lower combined thromboembolic and bleeding complications than both INR greater than 3.4 and INR less than 2.0. The explanation for the lower INR target may be related to a higher bleeding event and intracerebral hemorrhage in Asian population as compared to Caucasians [26]. When we consider the INR level that is most suitable for the balance of risk and benefit ratio towards a lower target than the general recommendation.

This study has several mentionable limitations. First and consistent with the retrospective nature of this study, some patient data may have been missing or incomplete. For example, some physicians or patients may have overlooked or ignored some episodes of minor bleeding, so those episodes would not have been documented. Second, the size of the study population was relatively small. Although we ran power analysis to calculate sample size needed for the primary objective of this study, we felt that the number of patients of 200 is relatively small. Further study with a larger number of study population may be needed to confirm the results of this study. Third, the patients enrolled in this study were from a single center. The nature of hospital and medical practice may be different. Also our center is Thailand’s largest tertiary referral hospital, which means that we are often referred patients with complicated and intransigent conditions. As such, it is possible that our findings may not be generalizable to patients with the same condition in other settings. Lastly, since we excluded patients with history of stroke or bleeding, we did not have data on optimal INR for this group which are considered a high risk group. We excluded patients with history of thromboembolic event and bleeding due to some reasons. For patients with history of stroke, most of them had some degree of residual neurological deficit. This could lead to problem of the documentation of recurrent stroke. For patients with history of bleeding, they could have some underlying disease that prone to recurrent bleeding even without warfarin. Moreover, many patients with history of stroke of bleeding may be a referred case from other hospital and may be difficult for the investigators to collect the data on the time relation of anticoagulant use and the onset of stroke or bleeding. Further prospective study in a larger study population is needed to confirm the findings of this study, and to narrow the optimal INR range in patients with certain demographic and clinical profiles.

## Conclusion

The optimal INR level was within the range of 2.0 to 3.4 in our cohort of Thai mechanical mitral valve replacement patients. This INR target may also be applied in Asian population. This suggestion should allow more room for INR adjustment which could avoid bleeding event in patients with mechanical mitral valve without losing the benefit of warfarin.

## Abbreviations

INR: International normalized ratio
LA: Left atrium
LVEF: Left ventricular ejection fraction
TIA: Transient ischemia attack
